# Post-marketing safety signals of Wilson’s disease therapies: evidence from FAERS and VigiBase

**DOI:** 10.1186/s13023-026-04310-9

**Published:** 2026-03-09

**Authors:** Hülya Tezel-Yalçın, Nadir Yalçın, Karel Allegaert, Pınar Erkekoğlu

**Affiliations:** 1 Department of Pharmaceutical Toxicology, Faculty of Pharmacy, Hacettepe University, Ankara, Türkiye 06230 Turkey; 2 Department of Clinical Pharmacy, Faculty of Pharmacy, Hacettepe University, Ankara, Türkiye 06230 Turkey; 3 Clinical Pharmacology and Pharmacotherapy, Department of Pharmaceutical and Pharmacological Sciences, KU Leuven, Leuven, 3000 Belgium; 4 Department of Development and Regeneration, KU Leuven, Leuven, 3000 Belgium; 5 Department of Hospital Pharmacy, Erasmus MC, Rotterdam, 3015 GD The Netherlands

**Keywords:** Wilson’s disease, D-penicillamine, Trientine, Copper, Hepato-lenticular degeneration, FAERS, VigiBase, Pharmacovigilance

## Abstract

**Introduction:**

Wilson’s disease is a rare autosomal copper metabolism recessive disorder that requires lifelong pharmacological treatment. D-penicillamine and trientine are the most commonly used copper chelators. However, their post-marketing safety reporting patterns remain insufficiently characterized.

**Methods:**

We conducted a pharmacovigilance study using the U.S. Food and Drug Administration Adverse Event Reporting System (FAERS) and the World Health Organization’s VigiAccess database. Because FAERS allows case-level signal detection whereas VigiAccess provides only aggregated data, analyses were intentionally restricted to parallel signal characterization, precluding event-matched or head-to-head comparisons. To reduce indication bias and ensure disease-specific assessment, FAERS analyses were restricted to reports explicitly listing “hepato-lenticular degeneration” as the indication for use. Temporal, geographical, and demographic trends were evaluated, and disproportionality analyses—including the reporting odds ratio (ROR), proportional reporting ratio (PRR), information component (IC), and multi-item gamma-Poisson shrinker (MGPS)—were performed to identify and validate adverse drug event (ADE) signals.

**Results:**

A total of 641 cases were retrieved from FAERS and 4,560 from VigiAccess. Trientine accounted for most cases in FAERS, whereas D-penicillamine predominated in VigiAccess. Temporal analysis showed earlier reporting peaks for D-penicillamine in VigiAccess, while trientine displayed a progressive rise after 2000. Regional differences were evident, with Europe dominating VigiAccess reports and Asia/America contributing more strongly to FAERS. Disproportionality analyses revealed significant signals: trientine was associated with hepatic failure, abdominal pain, tremor, nausea, and fatigue, while D-penicillamine demonstrated signals for dystonia, arthritis, tremor, and nausea. Extremely high estimates for hepato-lenticular degeneration likely reflect confounding by indication.

**Conclusion:**

Our findings characterize drug-specific safety reporting patterns for copper chelators used in Wilson’s disease within two complementary pharmacovigilance systems. Rather than establishing relative safety, the observed differences reflect distinct reporting profiles shaped by database structure, regulatory context, and clinical use. Integrating national and international pharmacovigilance data provides complementary insights into post-marketing safety signals and may support tailored clinical monitoring strategies without implying head-to-head comparative risk.

**Supplementary Information:**

The online version contains supplementary material available at 10.1186/s13023-026-04310-9.

## Introduction

Wilson’s disease (hepatolenticular degeneration) is a rare autosomal recessive disorder of copper metabolism, with the majority of cases resulting from pathogenic variants in the *ATP7B* gene, which encodes a copper-transporting P-type ATPase essential for hepatic copper excretion [1]. This defect leads to progressive accumulation of copper in the liver, brain, and other organs, producing a heterogeneous clinical spectrum that includes hepatic dysfunction, neurological impairment, and psychiatric disturbances [2]. Without treatment, Wilson’s disease may be almost fatal [3]. However, early diagnosis and lifelong pharmacological therapy have markedly improved survival and quality of life [4, 5].

The cornerstone of therapy includes copper-chelating agents [e.g. D-penicillamine, triethylenetetramine (trientine), tetrathiomolybdate] or zinc, which reduce intestinal copper absorption [6]. D-penicillamine, the therapeutically active R-enantiomer of penicillamine, is primarily used in the treatment of Wilson’s disease at the doses of 750 to 1500 mg/day whereas the L-enantiomer is toxic and not applied clinically. It functions as a chelating agent by binding heavy metals such as copper, lead, and mercury, and forms soluble complexes that are excreted in urine. Through disulfide bond formation with cysteine, it enhances urinary solubility and prevents cystine stone formation [7]. Trientine is a polyamine-like compound that chelates copper by coordinating its four nitrogen atoms to form a stable planar ring complex, which is then excreted in the urine. It received approval for clinical use in the United States in 1969 and is formally indicated for the treatment of Wilson’s disease, particularly in patients who are intolerant to D-penicillamine [8].

These drugs have transformed Wilson’s disease from a lethal disorder into a manageable chronic condition. Nevertheless, their use is frequently complicated by adverse drug events (ADEs) [6]. D-penicillamine is associated with dermatological, renal, and autoimmune complications, and in some cases paradoxical neurological worsening [6, 7]. While generally better tolerated, trientine may cause hematological toxicity, gastrointestinal intolerance, and hypersensitivity [8]. Because treatment is lifelong and often initiated early life [3], ensuring the safety and tolerability of these drugs is crucial [9]. Moreover, ADEs may adversely influence medication adherence, which is already a challenge in pediatric and adolescent patients with Wilson’s disease, thereby potentially compromising long-term treatment outcomes [10].

Despite their widespread use, systematic data on ADEs related to Wilson’s disease therapies remain scarce. This rare disease limits the feasibility of large randomized controlled trials [2], and most evidence is derived from case reports, small series, or single-center observational cohorts [9]. Consequently, the full spectrum and relative frequencies of ADEs remain poorly defined, leaving clinicians reliant on limited evidence when balancing therapeutic efficacy and safety [2].

Pharmacovigilance databases provide a unique opportunity to address this gap [11]. The U.S. Food and Drug Administration Adverse Event Reporting System (FAERS) and the World Health Organization’s global database of individual case safety reports (VigiBase) collectively contain millions of spontaneous reports submitted by healthcare professionals and patients worldwide [12]. These repositories enable the detection of disproportionate reporting and the identification of potential safety signals in real-world clinical practice, particularly for rare diseases where conventional evidence is limited. By aggregating reports from diverse populations and healthcare systems, pharmacovigilance data complement clinical trial evidence and broaden the understanding of post-marketing drug safety [13]. Importantly, pharmacovigilance databases such as FAERS and VigiAccess are not designed to support direct or indirect comparative safety assessments between therapies. Differences in data granularity, reporting practices, and regulatory contexts preclude event-matched or head-to-head analyses across drugs. Accordingly, the present study does not aim to establish relative safety or to inform therapeutic choice. Instead, it focuses on characterizing drug-specific post-marketing safety reporting patterns within and across national and international pharmacovigilance systems, with the goal of enhancing signal awareness and supporting hypothesis generation for future targeted studies. For broader evaluation of reporting patterns and support the consistency of the identified safety signals, four complementary disproportionality methods were applied: Reporting odds ratio (ROR), proportional reporting ratio (PRR), Bayesian confidence propagation neural network (BCPNN), and the multi-item gamma-Poisson shrinker (MGPS) [14].

## Material and method

### Study design

We conducted an observational, cross-sectional analysis of the FAERS and VigiBase databases to investigate ADEs associated with D-penicillamine and trientine. Given the use of anonymized secondary data, formal ethical approval was not required.

### Data extraction from FAERS and VigiBase databases

For the FAERS dataset, reports were retrieved using the following active pharmaceutical ingredients: “D-penicillamine,” “penicillamine,” “penicillamine hydrochloride,” “trientine,” “trientine hydrochloride,” and “trientine tetrahydrochloride.” Data extraction from the FAERS database was conducted on 23 August 2025, spanning from the database’s inception in 1968. Records corresponding to these active ingredients were merged. A multi-step filtration process was applied to ensure the safety signals were specifically relevant to the rare disease focus of this study. Initially, 1,714 reports were identified for D-penicillamine and 396 for trientine. Reports were then excluded if they lacked a specified indication or were associated with non-Wilson conditions (e.g., rheumatoid arthritis, cystinuria, or lead poisoning) [7]. To minimize potential “clinical noise”, the final analysis was restricted to cases where “hepato-lenticular degeneration” was the confirmed indication and the drug was identified as the primary suspect, resulting in a refined cohort of 257 cases for D-penicillamine and 384 cases for trientine.

For VigiBase, data were accessed through its publicly available interface, VigiAccess. Unlike FAERS, VigiAccess does not allow direct case-level data extraction. Instead, it provides aggregated information including the number and percentage of reported ADEs and available demographic data. Therefore, analyses using VigiAccess were limited to descriptive statistics only based on aggregated counts. A total of 4,560 cases were identified (4,005 D-penicillamine and 555 trientine). It is important to emphasize that while FAERS permits granular, case-level data extraction suitable for rigorous disproportionality analyses, VigiAccess serves as a public interface providing only aggregated summary statistics. Consequently, the data architecture of VigiAccess precludes the calculation of signal detection indices and restricts the analysis to descriptive reporting trends and frequency distributions. Due to the lack of case-level detail and fundamental differences in database stracture, direct event-matched comparative analyses were not feasible.

### Data analysis

The study followed the recommendations of the “REporting of a Disproportionality analysis for drUg Safety signal detection using individual case safety reports in PharmacoVigilance (READUS-PV)” statement. For FAERS data, signal detection was conducted using ROR, PRR, and BCPNN in line with internationally recommended methodologies for signal detection. These formulas and interpretive criteria are summarized in the Supplementary Material. In addition, Empirical Bayes Geometric Mean (EBGM) values derived from the MGPS model were calculated as a supplementary sensitivity analysis to account for potential instability in low-count cells, with detailed estimates provided in the Supplementary Material.

For the detailed disproportionality and descriptive analyses, a frequency-based selection strategy was used to identify the top five adverse drug events (ADEs) for each chelator. Specifically, preferred terms (PTs) with the highest absolute reporting counts in the FAERS and VigiAccess databases were prioritized for in-depth signal detection, ensuring that the analysis focused on the most frequently reported safety concerns reflected in real-world reporting patterns. As expected, this approach yields distinct ADE sets for each chelator and allows the characterization of each drug to be grounded in its most frequent and potentially clinically relevant safety signals.

For VigiAccess data, only descriptive analyses of ADE frequencies and distributions were performed. To further explore reporting patterns, exploratory logistic and Poisson regression models were applied, with age and sex selected as primary covariates due to their consistent availability across both databases.

Overall, the analytical strategy was intentionally designed as a parallel, drug-specific signal characterization rather than an event-matched or head-to-head comparative analysis. The frequency-based selection of adverse drug events was intended to delineate salient post-marketing safety signals and to define each chelator’s safety reporting footprint within its respective pharmacovigilance context, rather than to directly compare or rank adverse event risks between therapies. This approach aligns with the descriptive and hypothesis-generating nature of spontaneous reporting systems and prioritizes interpretability while acknowledging the structural constraints of the underlying databases.

## Results

All results are presented descriptively to characterize drug-specific reporting patterns within pharmacovigilance databases and are not intended to support direct or indirect comparative safety interpretations.

**Fig. 1:**
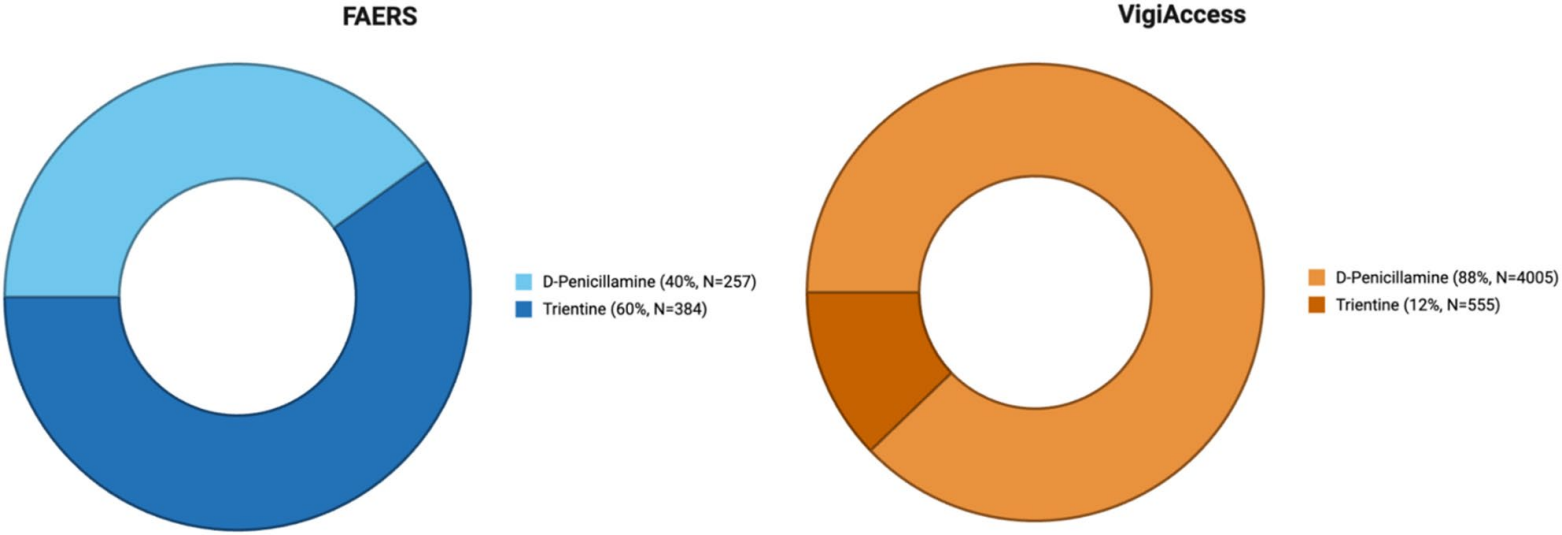
Distribution of D-penicillamine and trientine case reports in FAERS and VigiAccess. *Note: Data represent absolute reporting counts and proportions within the FAERS/VigiAccess database. These values are not adjusted for total drug exposure or prescription volume and should be interpreted as reflecting reporting patterns rather than absolute clinical risk or incidence rates

In FAERS, 641 cases were identified and in VigiAccess 4560 cases were recorded, covering D-penicillamine and trientine. Within FAERS, a greater proportion of reports involved trientine, whereas in VigiAccess a larger share of reports concerned D-penicillamine (Fig. 1).

**Fig. 2:**
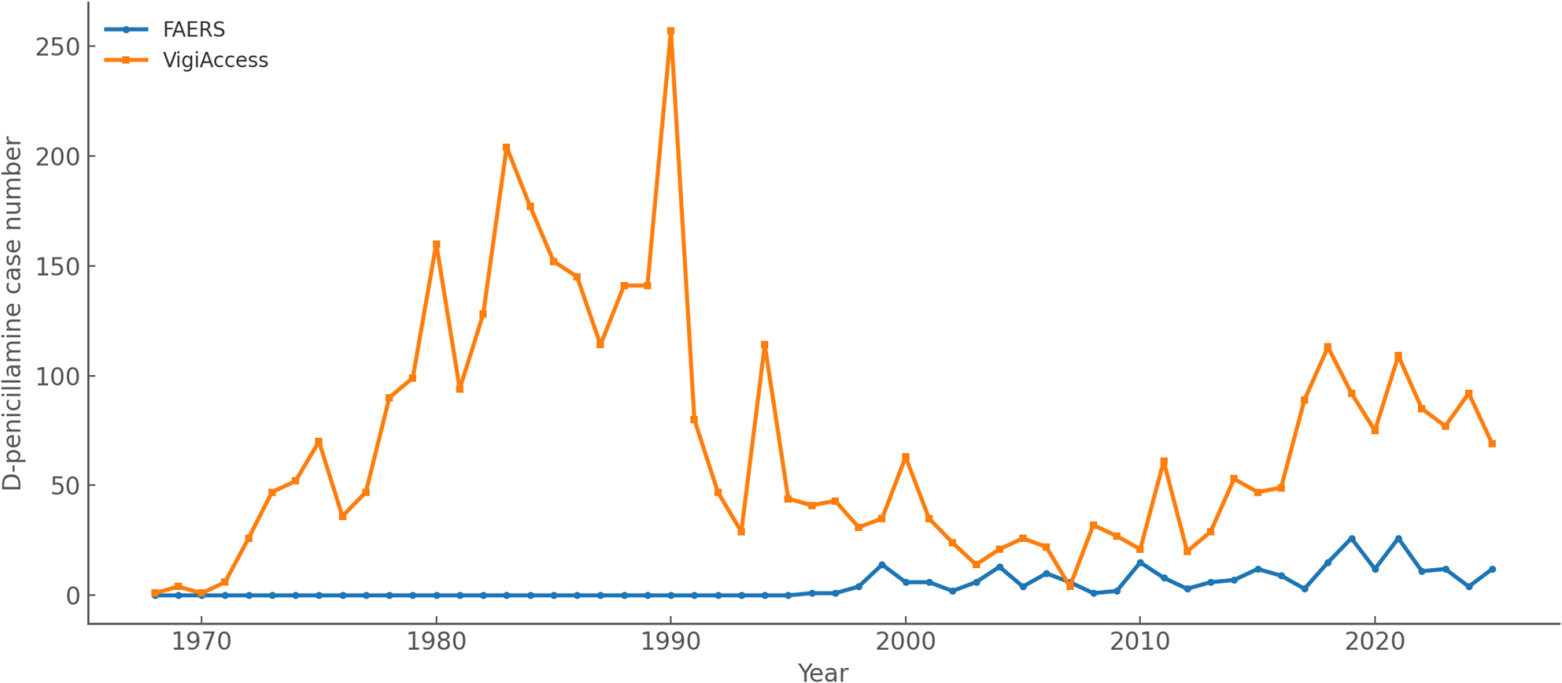
D-penicillamine reporting trends over the years

In FAERS, no cases of D-penicillamine were reported until the mid-1990s, after which the number of reports gradually increased with fluctuations. Peaks were observed in 1999 (14 cases), 2010 (15 cases), 2019 (26 cases), and 2021 (26 cases). In VigiAccess, reports were already present in the early 1970s, with a progressive rise during the following decades and a maximum of 257 cases in 1990. After 2000, the number of cases remained variable, with further increases observed in 2018 (113 cases), 2019 (92 cases), and 2021 (109 cases). D-penicilliamine reporting trends in years between 1968 and 23 August 2025 are shown in Fig. 2.

**Fig. 3:**
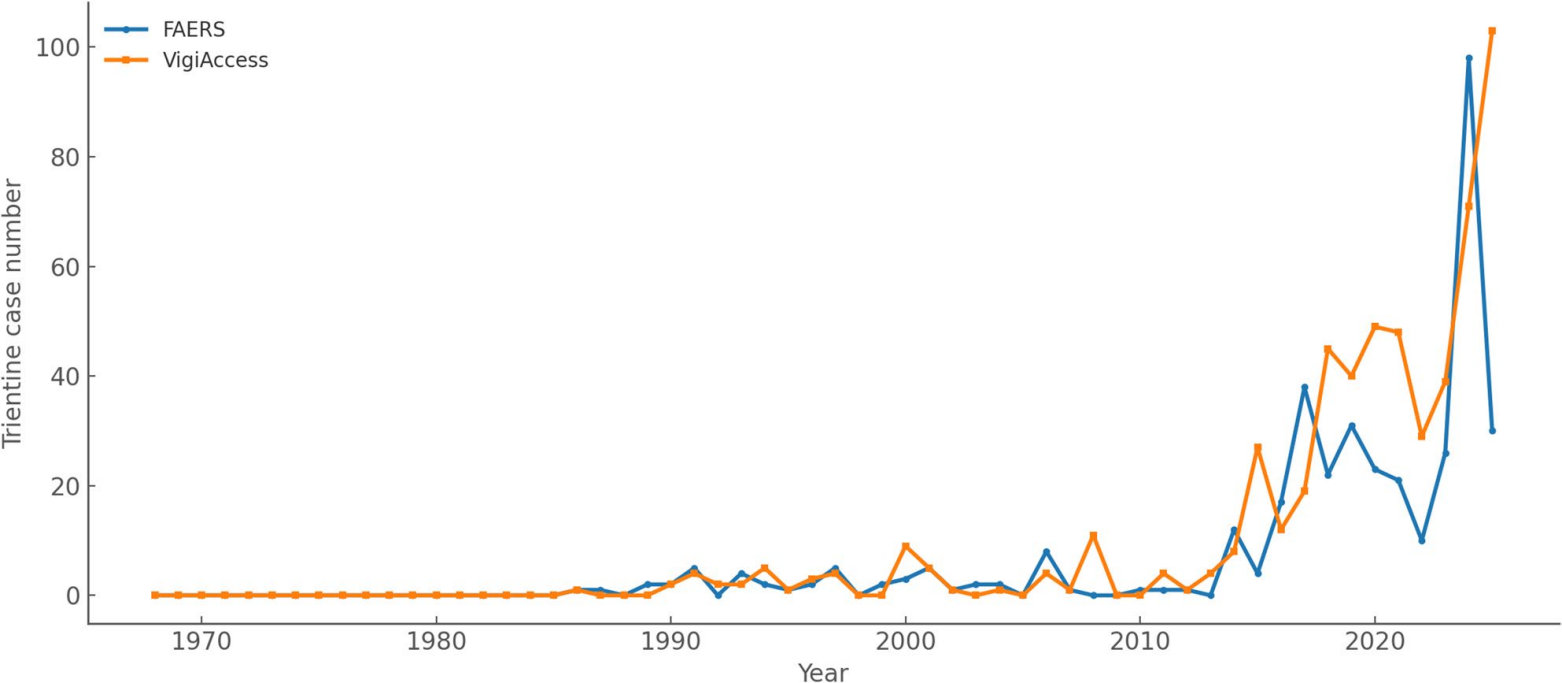
Trientine reporting trends over the years in FAERS and VigiAccess

For trientine, both FAERS and VigiAccess contained no or very few cases before the late 1980s. In FAERS, the number of reports increased after 2000, with a maximum of 98 cases in 2024. In VigiAccess, the number of reports began to increase from the early 1990s onwards, reaching higher levels than FAERS in most years. The maximum was recorded in 2025 with 103 cases. Trientine reporting trends in years between 1968 and 23 August 2025 are shown in Fig. 3.

According to the time trend analysis, across years FAERS reports increased for both drugs. However, the annual growth rate of reports differed between drug-specific reporting series. In Poisson models, the incidence-rate ratio (IRR) per calendar year was 1.03 for penicillamine (IRR 1.035, 95% CI 1.019–1.051, *p* = 1.1 × 10⁻⁵) and 1.12 for trientine (IRR 1.119, 95% CI 1.104–1.135, *p* < 1 × 10⁻⁵⁸). The Year×Drug interaction was significant (β = 0.079, *p* = 6.0 × 10⁻¹⁴), indicating heterogeneity in temporal reporting dynamics across drug-specific report sets. When counts were aggregated by period, trientine exceeded penicillamine both before and after 2000 (Pre-2000: 26 vs. 19; 2000+: 348 vs. 228, respectively).

**Fig. 4:**
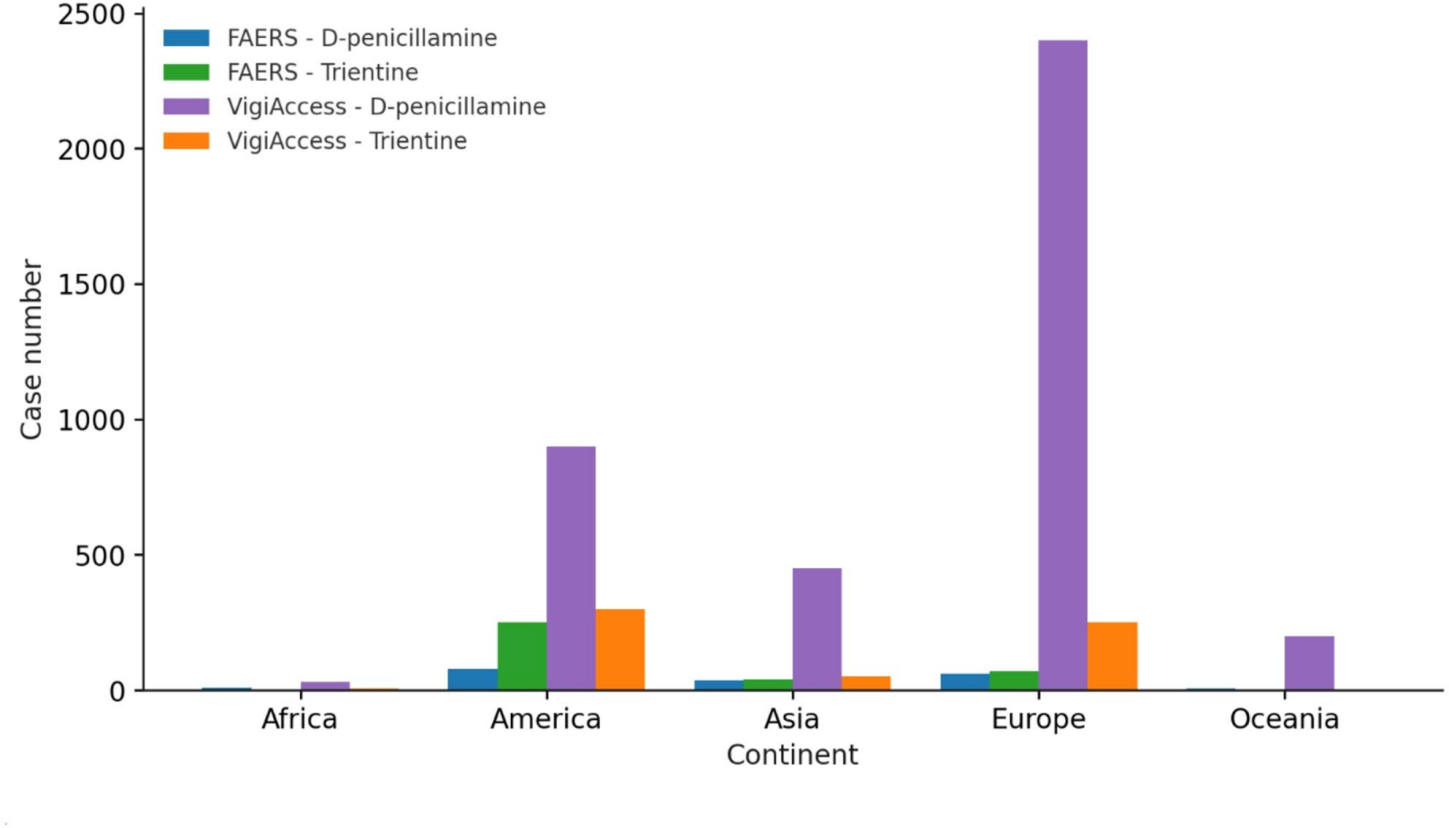
Case distribution by continent in FAERS and VigiAccess for D-penicillamine and trientine

In FAERS, most cases originated from Asia with 325 reports and from both North and South America with 324 reports, followed by 115 cases from Europe, while only 4 and 1 cases were reported from Oceania and Africa, respectively. In VigiAccess, Europe accounted for the majority with 2646 cases, followed by 1194 from the both North and South America and 481 from Asia, whereas Oceania and Africa contributed 218 and 21 cases, respectively (Fig. 4).

**Fig. 5:**
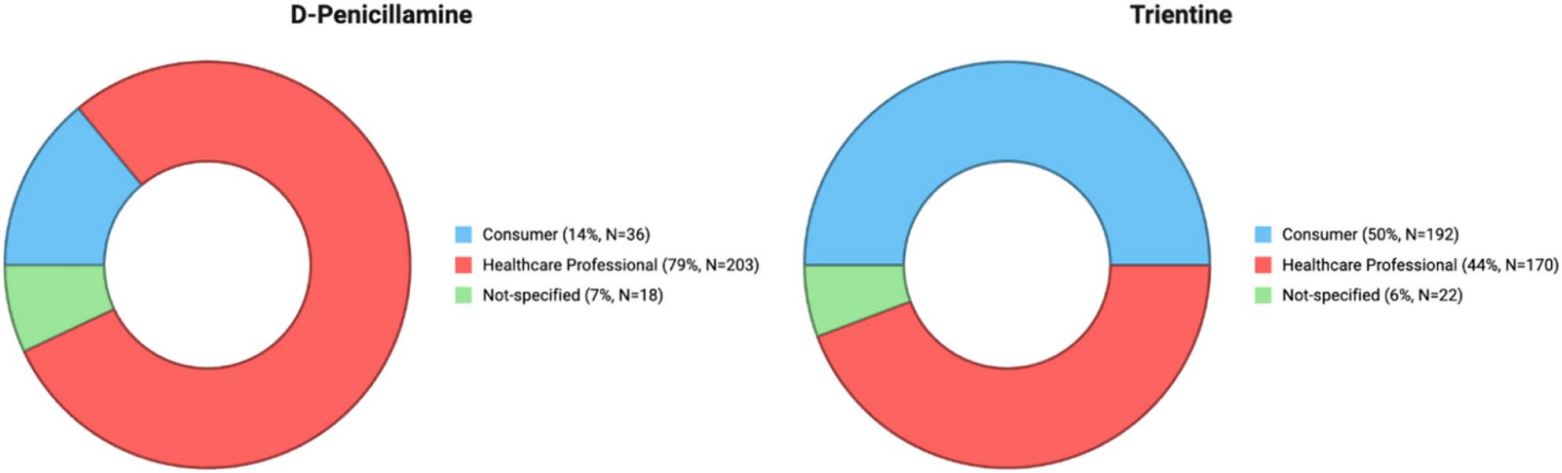
Distribution of case reports by reporter type for D-penicillamine and Trientine in FAERS

Reporting source distributions differed between the two drugs, with healthcare professionals accounting for a larger proportion of D-penicillamine reports, while consumer reports constituted a substantial share for trientine as presented in Fig. 5.

**Fig. 6:**
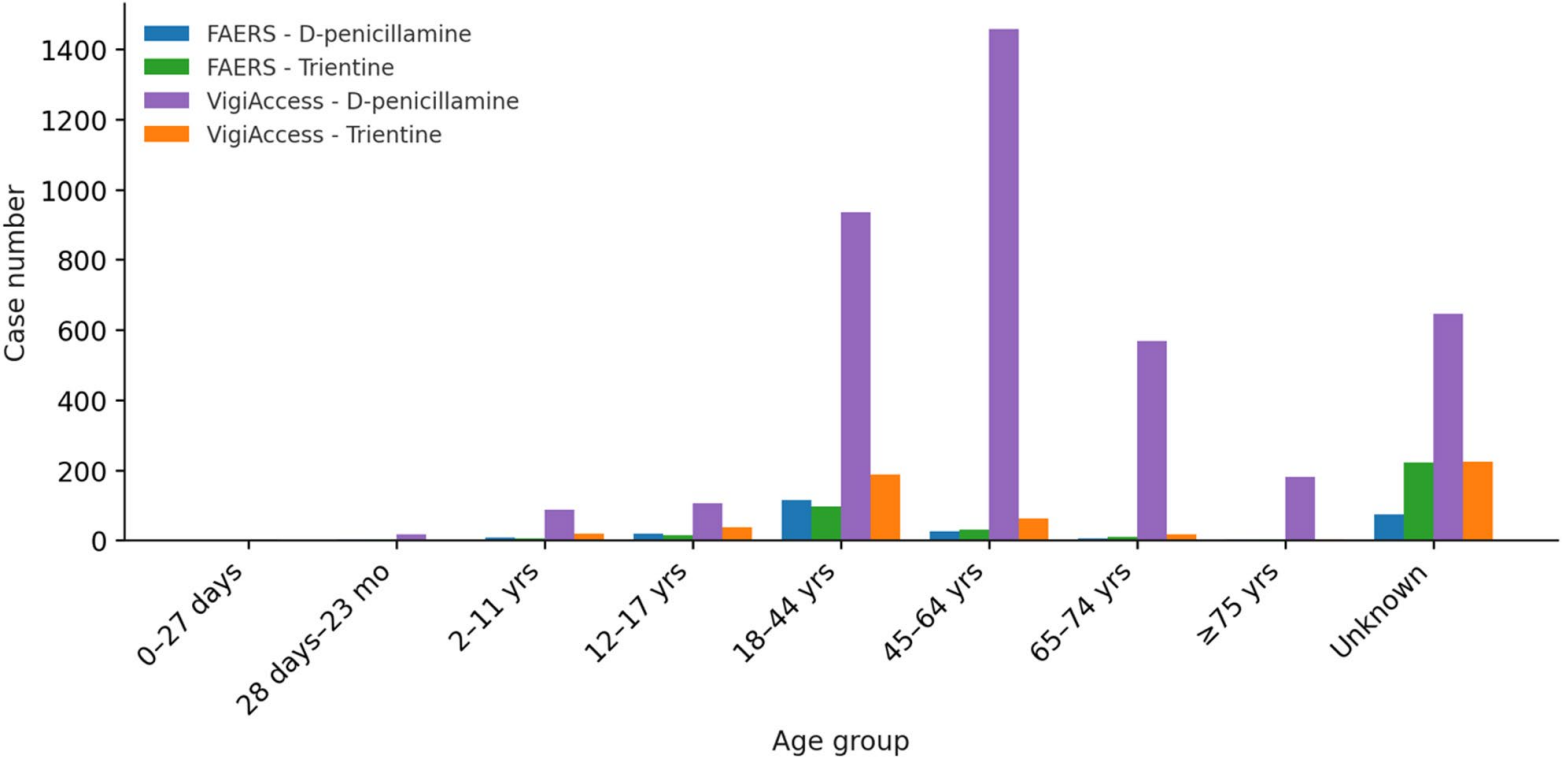
Distribution of case reports for age groups for D-penicillamine and trientine in FAERS and VigiAccess

In both databases, the majority of cases were reported in adult age groups, with the highest frequencies observed between 18 and 64 years, while pediatric and elderly cases were relatively less as represented in Fig. 6.

**Fig. 7:**
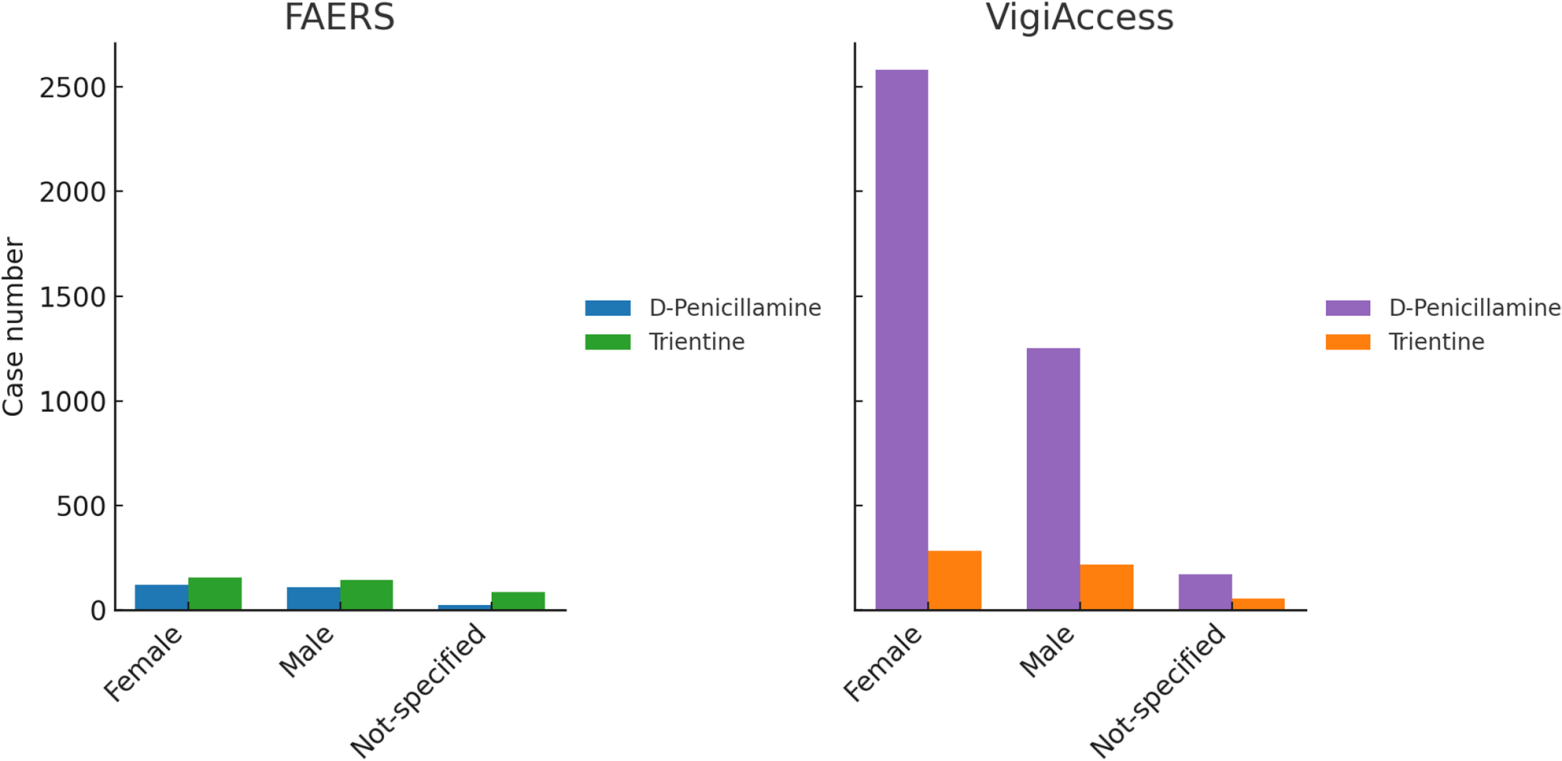
Gender-based reporting patterns of D-penicillamine and trientine across FAERS and VigiAccess

The gender distribution of adverse event reports is shown in Fig. 7. Gender distribution in FAERS is relatively balanced between females (277) and males (253), while a considerable number of cases were classified as not specified (111). In contrast, VigiAccess was dominated by female reports (2863), followed by males (1470), with 227 cases lacking gender specification.

**Fig. 8:**
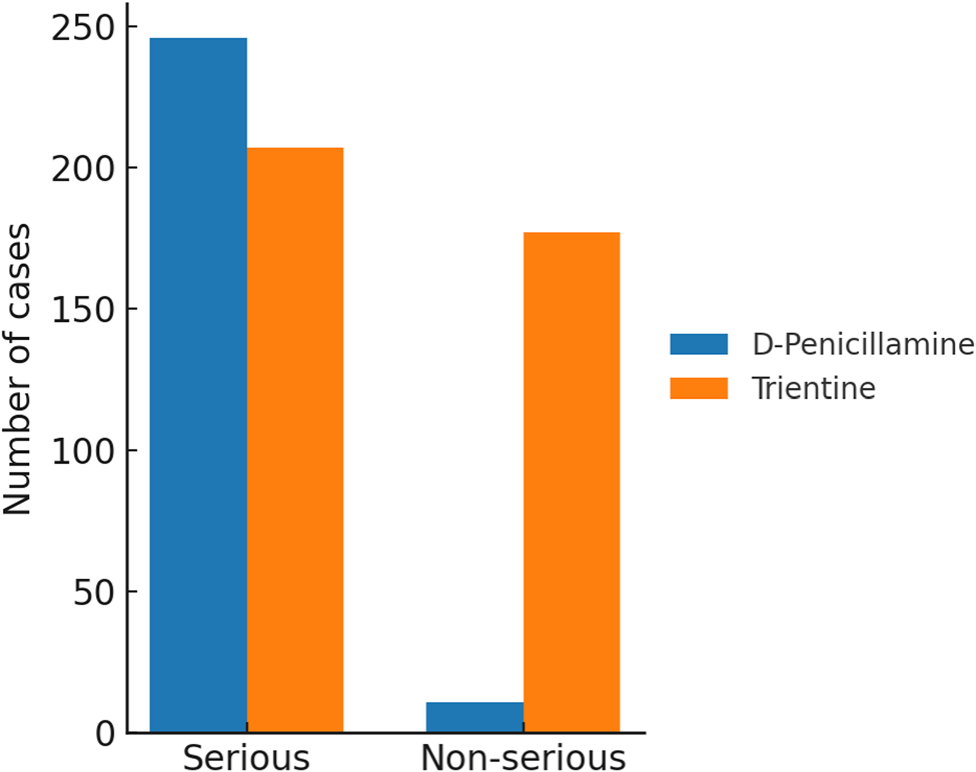
Proportional distribution of serious *versus* non-serious reports associated with D-penicillamine and trientine in FAERS. *Note: Data represent absolute reporting counts and proportions within the [FAERS/VigiAccess] database. These values are not adjusted for total drug exposure or prescription volume and should be interpreted as reflecting reporting patterns rather than absolute clinical risk or incidence rates

In total, 641 reports were identified. Within the FAERS database, reports involving D-penicillamine were more frequently classified as serious, whereas reports involving trientine exhibited a broader distribution across seriousness categories. In contrast, trientine demonstrated a different distribution pattern, with 207 serious and 177 non-serious reports (Fig. 8).

A chi-square test was performed to assess the association between drug type and report seriousness. The analysis revealed a statistically significant difference (χ² = 127.86, df = 1, *p* < 0.0001), indicating statistically significant heterogeneity in seriousness classification across drug-specific report sets. Reports for D-penicillamine were predominantly classified as serious (246 out of 257; 95.7%), whereas reports for trientine were characterized by a larger proportion of non-serious regulatory outcomes (177 out of 384; 46.1%). Statistical significance in this context reflects differences in reporting distributions rather than evidence of differential clinical risk between therapies.

**Fig. 9:**
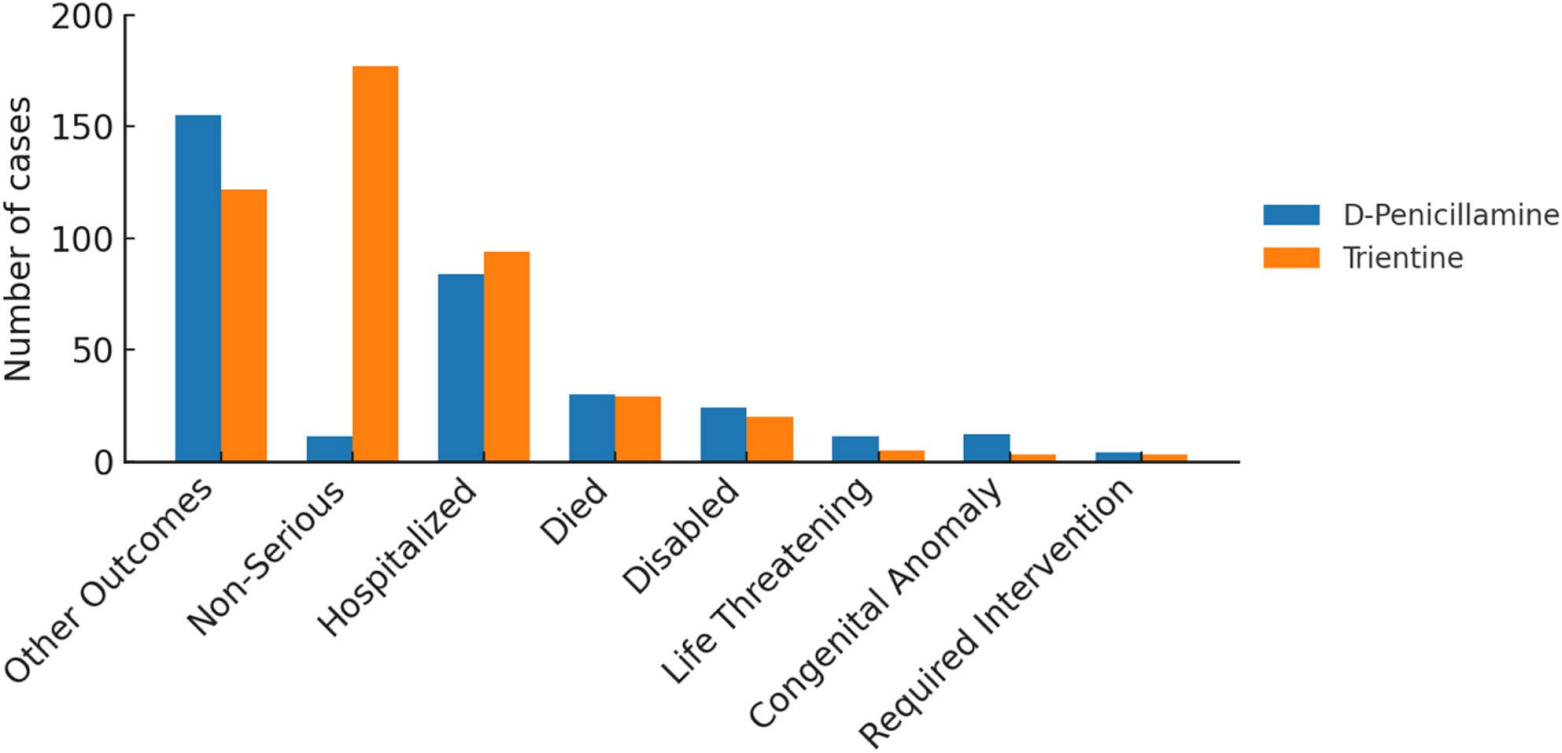
Distribution of reported outcomes for D-penicillamine and trientine

Among the reported outcomes, “other outcomes” represented the largest category for both drugs, accounting for 155 cases with D-penicillamine and 122 cases with trientine. Non-serious regulatory outcomes were more frequently reported within trientine-associated case sets (177 vs. 11). Hospitalization was reported in 94 trientine-associated cases and in 84 cases involving D-penicillamine, while death was reported in 29 and 30 cases, respectively. Congenital anomalies and life-threatening events were reported within D-penicillamine-associated cases, while disability and intervention-required outcomes were infrequently reported for both drugs (Fig. 9).

In the binary logistic regression for seriousness, males were associated with lower odds of serious reporting versus females (OR 0.20, 95% CI 0.05–0.83, *p* = 0.026). In the hospitalization model, older age was associated with a higher likelihood of hospitalization (OR per year 1.03, 95% CI 1.00–1.06, *p* = 0.038). These exploratory models were intended to describe associations within reported cases and do not imply causal or comparative safety relationships.

### D-Penicillamine associated Top 5 ADEs in FAERS and VigiAccess

**Fig. 10:**
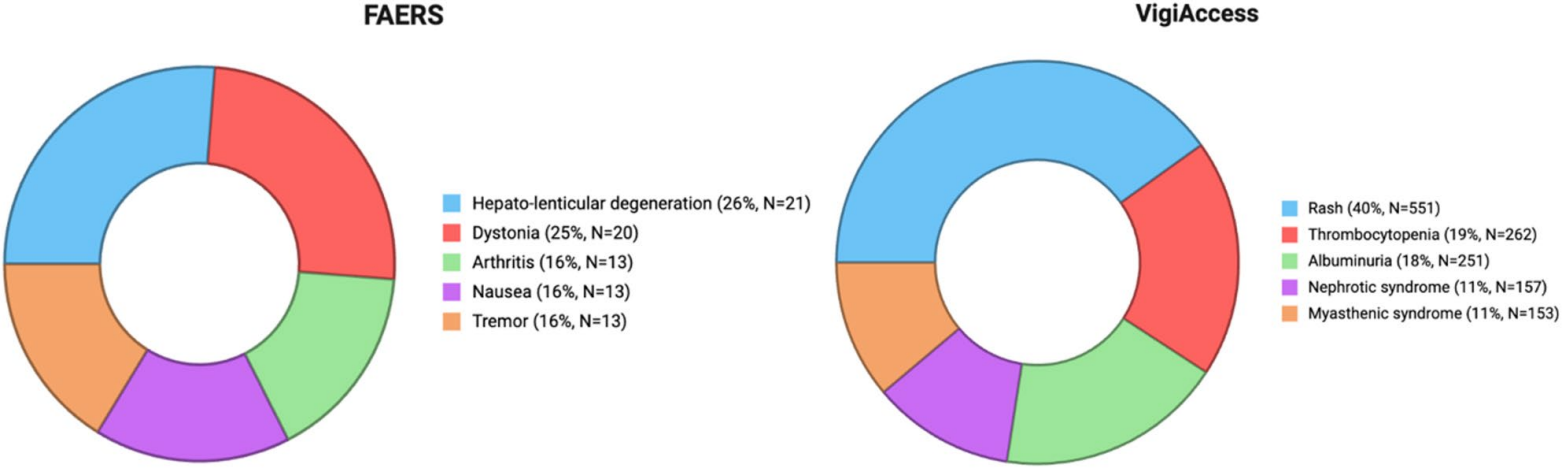
Most frequently reported ADEs for D-Penicillamine in FAERS and VigiAccess

Hepato-lenticular degeneration, dystonia, arthritis, nausea, and tremor emerged as the most frequently reported ADEs associated with D-penicillamine in FAERS, whereas rash, thrombocytopenia, albuminuria, nephrotic syndrome, and myasthenic syndrome were predominant in VigiAccess. While the absolute numbers differed between the two databases, the overall ranking of adverse events showed database-specific patterns (Fig. 10).

Disproportionality analyses of D-penicillamine showed clear signals for dystonia, arthritis, and tremor. Nausea also exceeded conventional thresholds but with a lower strength of association. Hepato-lenticular degeneration produced very high disproportionality estimates. Detailed values are presented in Table 1.

**Table 1:** Disproportionality measures (ROR, PRR, and IC) for the top five adverse events associated with D-Penicillamine, based on FAERS data

Adverse event	ROR (95% CI)	PRR	IC	IC₀.₂₅	EBGM (EB05–EB95)
Nausea	2.01 (1.15–3.52)	1.96	0.97	0.21	1.836 (0.821–4.105)
Tremor	9.12 (5.22–15.94)	8.71	3.12	2.36	5.618 (0.479–65.916)
Dystonia	117.0 (78.7–174.0)	106.7	6.64	6.17	17.715 (0.031–10039.025)
Arthritis	28.6 (16.2–50.3)	27.1	4.76	4.19	8.491 (0.205–350.987)
Hepato-lenticular degeneration	2.32 × 10⁴ (1.44 × 10⁴–3.76 × 10⁴)	2.13 × 10⁴	14.15	13.61	21.975 (0.010–47,729.904)

*ROR = Reporting Odds Ratio; PRR = Proportional Reporting Ratio; IC = Information Component; IC₀.₂₅ = lower 95% credibility bound; EBGM = Empirical Bayes Geometric Mean; EB05/EB95 = 90% interval bounds

### Trientine associated top 5 ADEs in FAERS and VigiAccess

**Fig. 11:**
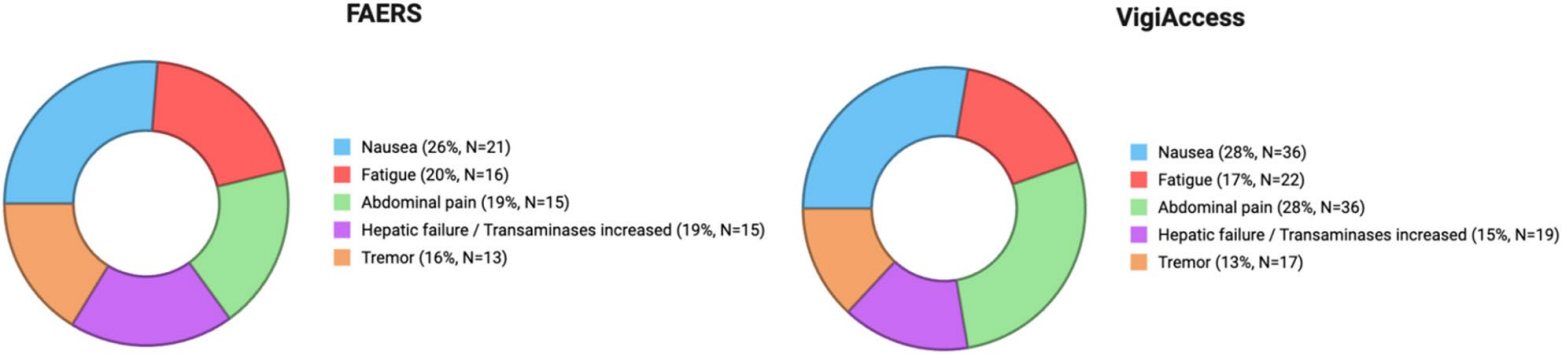
Most frequently reported ADEs for trientine in FAERS and VigiAccess

In both FAERS and VigiAccess databases, nausea, fatigue, abdominal pain, hepatic-related events, and tremor emerged as the most frequently reported adverse events associated with trientine. While the absolute numbers differed between the two databases, the overall ranking of ADE showed substantial overlap (Fig. 11).

Disproportionality analyses of trientine showed clear signals for nausea, fatigue, abdominal pain, hepatic failure and tremor. Detailed values are presented in Table 2.

**Table 2:** Disproportionality measures (ROR, PRR, IC and EBGM) for the top five adverse events associated with trientine, based on FAERS data

Adverse event	ROR (95% CI)	PRR	IC	IC₀.₂₅	EBGM (EB05–EB95)
Nausea	2.19 (1.41–3.39)	2.12	1.09	0.49	2.018 (0.997–4.088)
Fatigue	1.81 (1.10–2.99)	1.78	0.83	0.14	1.699 (0.864–3.339)
Abdominal pain	4.68 (2.79–7.84)	4.53	2.18	1.46	3.712 (0.810–17.009)
Hepatic failure	36.29 (21.66–60.83)	34.92	5.13	4.41	11.191 (0.114–1100.119)
Tremor	6.00 (3.45–10.43)	5.83	2.54	1.77	4.335 (0.648–28.992)

*ROR = Reporting Odds Ratio; PRR = Proportional Reporting Ratio; IC = Information Component; IC₀.₂₅ = lower 95% credibility bound; EBGM = Empirical Bayes Geometric Mean; EB05/EB95 = 90% interval bounds

ROR, PRR, and IC values exceeded conventional thresholds for nausea, fatigue, abdominal pain, hepatic failure, and tremor, with hepatic failure yielding the highest disproportionality estimates. The main disproportionality analyses based on ROR, PRR, and IC demonstrated consistent signal patterns across the top reported adverse events. EBGM estimates obtained through the MGPS model were generally concordant with these results, while providing more conservative values for events with sparse data.

## Discussion

Wilson’s disease (WD) is a rare autosomal recessive disorder of copper metabolism, and effective pharmacological therapy has transformed this disease from a fatal to a chronic condition with long-term survival [15, 16]. Among available therapies, D-penicillamine has historically been the mainstay, whereas trientine is more recently used as an alternative in patients intolerant to D-penicillamine. Despite their efficacy, both chelators are associated with ADEs that may compromise tolerability, adherence, and treatment outcomes [10]. A detailed understanding of their safety profiles, particularly through large-scale pharmacovigilance data, is crucial to inform clinicians where evidence on randomized trials is limited.

To address this evidence gap, our study focused on two of the most comprehensive and complementary pharmacovigilance datasets: FAERS and VigiBase. FAERS provides detailed, case-level information within the U.S. regulatory framework, while VigiBase encompasses first international reports from over 130 member countries. Analyzing both databases allowed us to capture national and global perspectives, thereby minimizing regional biases and enhancing the generalizability of our findings. In interpreting the results of this study, it is imperative to avoid asserting a definitive safety hierarchy or clinical superiority of one chelator over another. The observed differences in reporting patterns such as the higher proportion of serious outcomes for D-penicillamine and the prevalence of gastrointestinal signals for trientine must be viewed within the context of “channeling bias” and differing treatment lines. Because trientine is often utilized as a second-line option for patients who are de-coppered or intolerant to first-line agents, its reporting profile is inherently influenced by a different clinical stage of Wilson’s disease compared to D-penicillamine. Therefore, the identified safety signals characterize each drug’s reporting footprint rather than establishing a direct comparative safety advantage. Accordingly, the present discussion focuses on contextualizing reporting patterns rather than inferring comparative clinical superiority or therapeutic decision-making.

The number of cases identified in our study differs from some previous large-scale pharmacovigilance analyses [17, 18]. These differences primarily arise from methodological variations in inclusion criteria. While broader studies often include all adverse event reports regardless of the underlying indication to provide a global safety overview, our study prioritized high specificity for Wilson’s disease. By excluding cases with non-Wilson indications or missing indication data, we aimed to characterize a safety footprint more directly applicable to the clinical management of hepato-lenticular degeneration. This targeted approach highlights the importance of indication-specific filtering in rare disease research to avoid the dilution of safety signals by unrelated pathologies.

### Case distribution across databases

Our evaluation of FAERS and VigiAccess revealed marked differences in the volume and distribution of case reports associated with D-penicillamine and trientine. Trientine was more frequently reported in FAERS, whereas D-penicillamine predominated in VigiAccess, a pattern likely reflecting regional prescribing practices and historical treatment preferences rather than intrinsic differences in drug safety [7]. In the United States, trientine has increasingly been used in patients intolerant to D-penicillamine, which is consistent with recent FAERS reporting trends [9]. Importantly, these discrepancies should be interpreted within the structural and methodological context of the databases. FAERS analyses were restricted to reports explicitly indicating Wilson’s disease to ensure diagnostic specificity, which inherently limited the number of included cases. In contrast, VigiAccess aggregates global reports without indication-level filtering and spans a longer historical timeframe, with records dating back to the 1970s.

Differences in global drug availability and market penetration further contribute to the observed reporting patterns. D-penicillamine has remained the first-line therapy for Wilson’s disease worldwide for more than six decades, accounting for its extensive representation in VigiAccess. Conversely, trientine is a more recent alternative with limited accessibility in many regions due to regulatory and economic constraints. While disproportionality metrics such as ROR and PRR aim to reduce the influence of absolute reporting volume, underlying prescribing patterns continue to shape the safety landscape captured by spontaneous reporting systems. Accordingly, the observed reporting differences are best understood as reflections of utilization context and database architecture rather than evidence of comparative clinical safety.

The observed disparities in adverse event reporting between the FAERS and VigiBase databases may be fundamentally influenced by regional genetic diversity and complex genotype-phenotype correlations. Recent studies have emphasized that the spectrum of ATP7B mutations varies significantly across different geographical cohorts, directly impacting the clinical manifestations of Wilson’s disease. For instance, in a large Turkish cohort, p.H1069Q remains the most prevalent mutation, frequently associated with neurological symptoms [19], whereas specific variants like p.R778L are more dominant in East Asian populations [20]. Research has established that certain mutations, such as the c.2299insC found in Mediterranean countries, are strongly linked to hepatic phenotypes, while others like p.Ala1003Thr are more common in patients presenting with neurological involvement [21, 22]. Furthermore, evidence from real-life registries suggests that even common mutations can exhibit low penetrance, leading to a wide spectrum of clinical severity and varying therapeutic requirements between regional cohorts [23].

Given that the underlying phenotype whether predominantly hepatic or neurological modulates the patient’s susceptibility to certain adverse drug reactions, it is plausible that the safety profiles of chelators (D-penicillamine and trientine) exhibit regional variations based on these genetic backgrounds. While spontaneous reporting databases like FAERS and VigiBase lack individual-level genomic data to prove direct causality, the divergence in safety signals across these global registries likely reflects the heterogeneous genetic landscape of the Wilson’s disease population.

### Temporal reporting trends

Marked differences were observed in temporal reporting patterns across databases. For D-penicillamine, FAERS exhibited a delayed onset of reports with a marked increase after the mid-1990s and peaks in 2019 and 2021, whereas VigiAccess demonstrated earlier and more pronounced reporting beginning in the 1970s, with a peak in 1990. This divergence is consistent with the regulatory and historical availability of penicillamine, which was approved by the U.S. FDA in 1956 and has been in clinical use since 1960 [24, 25]. Its long-standing global use likely contributed to the earlier accumulation of international pharmacovigilance reports captured in VigiAccess. In contrast, trientine showed a later emergence in both databases. Although trientine dihydrochloride (Syprine^®^) received U.S. FDA approval in 1985 for patients intolerant to D-penicillamine, broader clinical uptake occurred more gradually, particularly outside the United States. Following 2000, trientine-associated reports increased progressively, reaching a maximum in VigiAccess in 2025 [26]. The more recent approval of trientine tetrahydrochloride in 2022 for Wilson’s disease patients [27] may have further influenced prescribing practices and subsequent reporting activity.

Importantly, these temporal patterns should be interpreted as reflections of reporting behavior rather than changes in intrinsic drug safety over time. Due to the absence of denominator data—such as total exposure or prescription volume—spontaneous reporting systems cannot support incidence-based risk estimation. Consequently, measures derived from time-series analyses, including incidence-rate ratios, indicate growth in reporting volume rather than true temporal changes in adverse event risk. These trends are likely shaped by extrinsic factors such as regulatory milestones, evolving clinical use, and increasing awareness, and therefore should be regarded as descriptive indicators of pharmacovigilance activity rather than causal evidence of emerging toxicity.

### Geographical variation

Geographical analyses highlighted further discrepancies. FAERS predominantly captured reports from Asia and the North and South America, whereas VigiAccess indicated a majority from Europe. This observation likely reflects Europe’s earlier and more widespread adoption of D-penicillamine, coupled with extensive pharmacovigilance monitoring infrastructure [28]. By contrast, the relatively low number of cases from Africa and Oceania across both databases suggest smaller patient populations. These findings underscore the importance of regional treatment practices, diagnostic availability, and healthcare infrastructure in shaping reporting patterns.

### Demographic factors

Age distribution showed a predominance of adult cases, consistent with long-term use of copper chelators for chronic management of Wilson’s disease [29]. Pediatric cases were comparatively underrepresented, which may relate not only to more cautious prescribing but also to delayed or missed diagnosis of Wilson’s disease in children. Literature consistently reports underdiagnosis in pediatric populations, which may underestimate ADEs [30]. Elderly patients were also less frequently reported, possibly due to competing comorbidities or shorter treatment durations. Gender analyses revealed more female cases in VigiAccess, which may reflect known clinical sex differences: women more susceptible to hepatic ADEs, whereas men more commonly develop neurological manifestations at younger ages [31, 32]. On the other hand, male sex was associated with a lower likelihood of serious ADE reporting compared to female. Such variations may affect reporting behaviors and highlight the necessity for sex- and age-specific pharmacovigilance efforts.

### Seriousness and outcomes

Analysis of regulatory seriousness classifications revealed differing reporting distributions for D-penicillamine and trientine within FAERS. Reports associated with D-penicillamine were more frequently categorized as serious, including outcomes such as hospitalization, congenital anomalies, and life-threatening events, whereas trientine-associated reports included a higher proportion of non-serious regulatory outcomes. These findings reflect how adverse events are reported and classified within spontaneous reporting systems rather than indicating intrinsic differences in clinical severity or toxicity between therapies.

It is essential to distinguish between the regulatory definition of seriousness used in pharmacovigilance and the clinical concept of severity. In FAERS and VigiAccess, seriousness is defined by predefined regulatory criteria—such as hospitalization, life-threatening events, or congenital anomalies—rather than by clinical intensity or long-term prognosis. Accordingly, the higher proportion of serious reports observed for D-penicillamine (95.7%) compared with trientine (53.9%) should be interpreted as a characteristic of reporting patterns within the database rather than as evidence of comparative clinical risk.

These distributions are likely influenced by reporting behavior and clinical context. Healthcare professionals may be more inclined to report events meeting regulatory seriousness criteria, particularly for long-established therapies such as D-penicillamine, which are subject to heightened clinical awareness and monitoring. Moreover, differences in patient populations, treatment lines, and underlying disease severity may further shape seriousness classifications. Consequently, seriousness-based findings in this study should be viewed as descriptive indicators of regulatory reporting outcomes rather than as measures of causal toxicity or therapeutic tolerability.

### Adverse events and disproportionality analysis

D-penicillamine remains a cornerstone in the treatment of Wilson’s disease; however, its initiation has occasionally been associated with paradoxical neurological deterioration. Disproportionality analyses from FAERS revealed strong signals for dystonia and tremor, while VigiAccess data additionally highlighted myasthenic syndrome. Clinical reports support these pharmacovigilance signals. Huang and Chu (1998) stated that Wilson’s disease patients who developed acute generalized dystonia and akinetic-rigid syndromes shortly after starting therapy showed corresponding thalamic and brainstem lesions on MRI. Svetel et al. (2001) reported a fatal case of *status dystonicus* occurring within weeks of treatment initiation, and Paliwal et al. (2010) documented cases of *status dystonicus* unresponsive to conventional antidystonic therapies but responsive to gabapentin. Beyond dystonia and tremor, several case reports detail D-penicillamine–induced myasthenic syndrome. Thapa et al. (2022) presented a pediatric case with fluctuating bulbar weakness compatible with myasthenia gravis six years within treatment period, while Antos et al. (2023) described an adult patient who developed ocular myasthenia gravis within 15 months of therapy. This situation resolved after discontinuation of D-penicillamine and initiation of symptomatic management. Mechanistically, D-penicillamine is hypothesized to alter acetylcholine receptor antigenicity or modulate immune presentation, thereby inducing autoantibody production. Taken together, these findings suggest that the spectrum of reported neurological adverse events associated with D-penicillamine spans from movement disorders to immune-mediated neuromuscular transmission abnormalities, highlighting the importance of vigilant clinical monitoring rather than establishing causal relationships [33].

Other ADEs of D-penicillamine are well-documented and involve hematologic, renal, musculoskeletal, gastrointestinal, and dermatologic systems [7]. Pharmacovigilance data from FAERS and VigiAccess consistently signal thrombocytopenia, albuminuria, nephrotic syndrome, arthritis, nausea, and rash. Similarly, cohort data in rheumatoid arthritis patients showed that rash, proteinuria, and thrombocytopenia are among the most frequent toxicities, often appearing within the first six months of treatment [34]. Renal involvement is particularly important: approximately 9% of patients developed proteinuria, usually within the first year, with many cases regressing after D-penicillamine discontinuation [35]. Case reports have further described nephrotic syndrome, sometimes with biopsy-proven membranous nephropathy, which resolved upon discontinuation of therapy [36–38]. Hematologic toxicity extends beyond simple thrombocytopenia, as rare but severe events such as thrombotic thrombocytopenic purpura were observed [39]. Dermatologic complications ranged from acute febrile eruptions shortly after initiation to chronic degenerative dermopathy with prolonged exposure [40]. In addition, immune-mediated arthritis has been recognized as a reversible adverse reaction, and nausea remains among the most common gastrointestinal complaints [7]. Collectively, these findings indicate a broad spectrum of reported systemic toxicities associated with D-penicillamine, underscoring the importance of structured laboratory and clinical monitoring. By contrast, the extreme signal for “hepato-lenticular degeneration” is best explained by confounding by indication, since this PT represents the underlying disease rather than a drug-induced event. Such artifacts emphasize the importance of careful interpretation of pharmacovigilance data and reinforce the descriptive nature of spontaneous reporting systems. The divergence in D-penicillamine’s safety signals between the two databases warrants a careful interpretation based on database architecture. In our study, FAERS data were strictly filtered for Wilson’s disease indications, which likely enhanced the detection of disease-specific neurological signals such as dystonia and tremor that might otherwise be diluted. Conversely, the higher prevalence of renal and hematological events (e.g., nephrotic syndrome and thrombocytopenia) in VigiAccess likely reflects the long-standing, established systemic toxicity profile of D-penicillamine across various indications, including rheumatoid arthritis, where such monitoring has been a clinical standard for decades. Furthermore, VigiAccess captures a longer historical period and a larger European cohort, whereas FAERS reflects more recent reporting patterns from North America. These discrepancies may be viewed as complementary; while VigiAccess provides a broad view of established systemic risks, FAERS offers insights into specific neurological outcomes within modern clinical contexts.

It is also important to note that the clinical manifestations and safety of D-penicillamine can be significantly influenced by dosage levels, titration strategies, and the clinical phenotype of Wilson’s disease (e.g., predominantly hepatic versus neurological). Furthermore, ADEs often cluster during the initial intensive de-coppering phase rather than long-term maintenance. However, due to the nature of spontaneous reporting in FAERS and VigiBase, critical details such as exact dosing, duration of therapy prior to the event, and baseline clinical presentation were frequently unavailable or incomplete. This lack of granular data precludes a definitive assessment of whether the higher frequency of ADEs for D-penicillamine is a result of intrinsic drug toxicity, high-dose regimens used in acute phases, or its application in specific high-risk phenotypes. Consequently, our findings should be interpreted with caution, and the observed reporting patterns should be viewed as hypothesis-generating signals requiring validation through clinical registries with more detailed patient histories.

For trientine, disproportionality signals were most pronounced for hepatic failure, abdominal pain, and tremor. Although clinical literature generally reports a favorable tolerability profile for trientine with respect to hepatic adverse events [41], the strong hepatic failure signal observed in our analysis warrants cautious interpretation. Trientine is frequently prescribed as a second-line therapy, particularly in patients who are intolerant to or have already developed complications under D-penicillamine treatment. As a result, individuals receiving trientine may represent a clinically more advanced subgroup with higher baseline hepatic vulnerability. This channeling of patients with greater disease severity introduces confounding by indication, whereby reported hepatic outcomes may reflect the natural progression of Wilson’s disease rather than a direct drug-induced hepatotoxic effect. Given the absence of granular clinical data—such as baseline liver function, disease stage, and treatment history—within spontaneous reporting systems, disproportionality analyses cannot fully disentangle drug-specific risks from underlying disease severity. Accordingly, the hepatic safety signals associated with trientine should be interpreted within the context of its clinical positioning and require confirmation in longitudinal studies with detailed phenotypic characterization. Gastrointestinal adverse events, particularly nausea and abdominal pain, were also consistently reported for trientine across both FAERS and VigiAccess. These findings align with previous clinical studies describing gastrointestinal tolerability issues as common, though generally manageable, adverse effects of trientine therapy [26, 42]. Fatigue, while non-specific, has similarly been reported as a constitutional symptom that may influence long-term adherence [42].

### Strengths and limitations

This study has several notable strengths. FAERS and VigiAccess were deliberately selected as they represent the largest and most accessible spontaneous reporting systems for independent pharmacovigilance research. FAERS enables detailed case-level analyses and quantitative signal detection, whereas VigiAccess, derived from the WHO global database, provides a broad international overview of reporting patterns. The combined use of these databases allowed us to integrate national and global perspectives, enhancing the contextual interpretation of adverse event reporting for D-penicillamine and trientine. Importantly, this approach enabled the evaluation of two therapies used in a rare disease such as Wilson’s disease, which would be challenging to study using conventional clinical registries. An additional strength lies in the application of multiple disproportionality methods, including frequentist (ROR, PRR) and Bayesian approaches (IC, MGPS), whose convergence increases confidence in the robustness of detected signals.

A key methodological distinction of this study relates to data structure. While FAERS permits case-level disproportionality analyses, VigiAccess provides only aggregated data, limiting analyses to descriptive reporting patterns. As a result, although VigiAccess contributed the majority of global reports, signal strength indices and detailed phenotypic correlations could only be assessed within FAERS. This structural asymmetry should be considered when interpreting the results and highlights the need for broader access to granular global pharmacovigilance data in future research.

It is essential to emphasize that the analytical framework of this study was intentionally designed for complementary safety profiling rather than head-to-head comparative risk assessment. Given the substantial differences in clinical positioning, market longevity, prescribing volumes, and global availability of D-penicillamine and trientine, direct statistical comparison could introduce significant bias. Accordingly, we applied a parallel signal detection strategy to independently characterize the drug-specific safety footprints of each chelator within FAERS and VigiAccess. Identical adverse events observed for both agents were interpreted within their respective reporting contexts. This approach acknowledges the intrinsic limitations of spontaneous reporting systems and aims to inform future hypothesis-driven pharmacoepidemiological research rather than dictate direct clinical decision-making.

Several limitations must also be acknowledged. Spontaneous reporting systems are inherently affected by underreporting, reporting bias, incomplete case information, and potential duplication. A substantial proportion of reports lacked complete demographic data, particularly regarding age and sex. Furthermore, key clinical variables such as disease severity, dosage, treatment duration, and co-medications were frequently unavailable, resulting in potential residual confounding. Consequently, regression analyses and disproportionality signals should be regarded as exploratory and hypothesis-generating.

Finally, due to the absence of denominator data and clinical staging information, this study does not allow estimation of absolute risk, incidence rates, or causal relationships between drug exposure and adverse events. The findings should therefore be interpreted as descriptive characterizations of safety reporting patterns within global pharmacovigilance databases, serving as a foundation for future prospective clinical or pharmacoepidemiological studies.

### Future strategies and recommendations

Future strategies in pharmacovigilance for Wilson’s disease should prioritize the integration of multiple international databases to minimize regional biases and improve the robustness of signal detection. Linking pharmacovigilance data with clinical registries and electronic health records can further help to differentiate true ADEs from disease-related manifestations [43, 44]. Particular emphasis should be placed on vulnerable groups, including children and pregnant women, where evidence remains limited despite lifelong treatment requirements. The integration of biobank resources has also been suggested as a promising avenue to strengthen rare disease pharmacovigilance and improve translational outcomes [45–47]. It may also include the application of machine learning approaches, such as Random Forest or Gradient Boosting, to predict adverse events and derive feature importance scores, thereby identifying which variables most strongly contribute to serious outcomes or hospitalization—an approach not yet explored in the context of Wilson’s disease pharmacovigilance.

## Conclusion

In conclusion, this study provides a comprehensive evaluation of the safety profiles of D-penicillamine and trientine using two complementary pharmacovigilance databases, FAERS and VigiAccess. While notable differences in reporting frequency and regional distribution were observed, consistent safety signals emerged across both platforms. Disproportionality analyses identified key ADEs, with trientine most strongly associated with hepatic and gastrointestinal outcomes, and D-penicillamine linked to neurological, musculoskeletal, and teratogenic complications. These findings align with existing clinical knowledge and highlight the value of pharmacovigilance systems in characterizing drug-specific risk patterns, particularly for rare diseases where prospective safety studies remain limited. Importantly, rather than guiding direct treatment selections—which must remain individualized—our results underscore the necessity of optimizing clinical monitoring strategies based on identified risk profiles. Specifically, these signals advocate for rigorous neurological surveillance for patients on D-penicillamine and focused gastrointestinal and hepatic monitoring for those receiving trientine. Furthermore, this work emphasizes the importance of integrating international resources and advanced statistical methods to disentangle true drug-related events from confounding by indication. Together, these findings serve as a hypothesis-generating foundation for future prospective pharmacoepidemiological studies and clinical registries, ultimately offering insights that inform clinical oversight, regulatory monitoring, and future research priorities.

## Supplementary Information

Below is the link to the electronic supplementary material.

Supplementary Material 1

## Data Availability

The raw data supporting the conclusions of this article are available from the authors upon reasonable request.
