# Supplementary material for: Post-marketing safety signals of Wilson’s disease therapies: evidence from FAERS and VigiBase
Source: Orphanet J Rare Dis. 2026 Mar 9;21:153. doi: 10.1186/s13023-026-04310-9 (PMC13085612; doi:10.1186/s13023-026-04310-9)
Supplement: Supplementary file 1 — Supplementary Material 1 [file 13023_2026_4310_MOESM1_ESM.docx]

**Supplementary Material**

## **Table 1.** Overview of disproportionality analysis methods applied in pharmacovigilance signal detection

| **Method** | **Formula** | **Description / Interpretation** |
| --- | --- | --- |
| Reporting Odds Ratio (ROR) | ROR = (a/c) / (b/d) = (a × d) / (b × c) | A frequentist metric comparing the odds of reporting a specific adverse event (AE) for a given drug relative to all others. a = both drug and AE; b = drug without AE; c = AE without drug; d = neither. ROR > 1 with 95% CI excluding 1 indicates a signal of disproportionate reporting. |
| Proportional Reporting Ratio (PRR) | PRR = [a/(a+b)] / [c/(c+d)] | Estimates the proportion of AE reports among all reports for a specific drug compared with all others. PRR ≥ 2, χ² ≥ 4, and ≥ 3 cases suggest a potential signal (Evans et al., 2001). |
| Bayesian Confidence Propagation Neural Network (BCPNN) | IC = log2 [ P(Drug, Event) / (P(Drug) × P(Event)) ] | Bayesian approach developed by WHO-UMC. The Information Component (IC) measures the deviation between observed and expected reporting. IC > 0 (lower 95% CI > 0) indicates disproportionate reporting. Robust for rare AEs and sparse datasets. |
| Multi-Item Gamma Poisson Shrinker (MGPS) | EBGM = (E(N) + α) / (E + β) or equivalently EBGM = (N / E) / shrinkage toward prior mean | Empirical Bayes method estimating the Empirical Bayes Geometric Mean (EBGM). N = observed count; E = expected count; α, β = prior parameters. EB05 ≥ 2 indicates a possible signal; EB05/EB95 represent 90% credibility limits. Applied in FDA’s OpenVigil and Empirica Signal systems. |

*In the 2×2 contingency table: a = reports with the drug and the event; b = reports with the drug and other events; c = reports with the event and other drugs; d = reports with other drugs and other events.

**Table 2.** 2×2 contingency tables for the disproportionality analyses of trientine and the five most frequently reported adverse events in FAERS.

| **Adverse Event** | **Trientine Cases (a)** | **Trientine Other (b)** | **Other Drugs Cases (c)** | **Other Drugs Other (d)** | **Total** |
| --- | --- | --- | --- | --- | --- |
| **Nausea** | 21 | 363 | 821,778 | 31,053,400 | 31,875,562 |
| **Fatigue** | 16 | 368 | 747,640 | 31,127,538 | 31,875,562 |
| **Abdominal pain** | 15 | 369 | 274,740 | 31,600,438 | 31,875,562 |
| **Hepatic failure** | 15 | 369 | 35,661 | 31,839,517 | 31,875,562 |
| **Tremor** | 13 | 371 | 185,038 | 31,690,140 | 31,875,562 |

*Each table includes the number of cases with the event of interest reported with trientine (a), trientine without the event (b), other drugs with the event (c), and other drugs without the event (d). These data formed the basis for the calculation of ROR, PRR, and IC values.

**Table 3.** 2×2 contingency tables for the disproportionality analyses of D-penicillamine and the five most frequently reported adverse events in FAERS.

| **Adverse Event** | **D-Penicillamine Cases (a)** | **D-Penicillamine Other (b)** | **Other Drugs Cases (c)** | **Other Drugs Other (d)** | **Total** |
| --- | --- | --- | --- | --- | --- |
| Hepato-lenticular degeneration | 21 | 236 | 122 | 31,875,183 | 31,875,562 |
| Dystonia | 20 | 237 | 22,979 | 31,852,326 | 31,875,562 |
| Arthritis | 13 | 244 | 80,455 | 31,794,850 | 31,875,562 |
| Nausea | 13 | 244 | 821,807 | 31,053,498 | 31,875,562 |
| Tremor | 13 | 244 | 185,038 | 31,690,267 | 31,875,562 |

*Each table includes the number of cases with the event of interest reported with D-penicillamine (a), D-penicillamine without the event (b), other drugs with the event (c), and other drugs without the event (d). These data formed the basis for the calculation of ROR, PRR, and IC values.
